# The Difference in Nutrient Intakes between Chinese and Mediterranean, Japanese and American Diets

**DOI:** 10.3390/nu7064661

**Published:** 2015-06-09

**Authors:** Ronghua Zhang, Zhaopin Wang, Ying Fei, Biao Zhou, Shuangshuang Zheng, Lijuan Wang, Lichun Huang, Shuying Jiang, Zeyu Liu, Jingxin Jiang, Yunxian Yu

**Affiliations:** 1 Zhejiang Provincial Center for Disease Control and Prevention, Hangzhou 310051, China; E-Mails: rhzhang@cdc.zj.cn (R.Z.); bzhou@cdc.zj.cn (B.Z.); lchhuang@cdc.zj.cn (L.H.); 2 Department of Epidemiology & Health Statistics, School of Public Health, School of Medicine, Zhejiang University, Hangzhou 310058, China; E-Mails: zpwang7891@126.com (Z.W.); myhero33@zju.edu.cn (Y.F.); shuangshuangzheng@163.com (S.Z.); wanglijuan890408@163.com (L.W.); 783305906@qq.com (S.J.); yuzeliu106@163.com (Z.L.); xiaoding5891@126.com (J.J.); 3 Chronic Disease Research Institute, School of Public Health, School of Medicine, Zhejiang University, Hangzhou 310058, China

**Keywords:** Chinese diet, Mediterranean diet, Japanese diet, American diet, nutrient intake, macronutrients, micronutrients

## Abstract

Across countries, the predominant diets are clearly different and highly related with human health. Therefore, it is necessary to evaluate dietary nutrients between them. This study aimed to evaluate dietary nutrients in China and compare those between Chinese and Mediterranean (Italian), Japanese and American diets. Dietary intakes of 2659 subjects in south-east China, Zhejiang province, from 2010 to 2012, were estimated by three consecutive 24-h dietary recalls. The contribution of carbohydrate to total energy in Chinese subjects was lower than that in Japanese and American subjects, but higher than that in Italian subjects. However, the energy contribution from fat in Chinese subjects was higher than that in Japanese and American subjects, and similar to that in Italian subjects. Moreover, the Chinese diet had lower daily intakes of fiber, calcium, phosphorus, potassium, selenium, vitamin A, vitamin B_1_, vitamin B_2_ and vitamin C, compared with the Japanese, American and Italian diets. Nevertheless, intakes of sodium, iron, copper and vitamin E were higher among Chinese people relative to the people of other three countries. The present study demonstrated that the structure of the Chinese diet has been shifting away from the traditional diet toward high-fat, low-carbohydrate and low-fiber diets, and nutrients intakes in Chinese people have been changing even worse than those in American people.

## 1. Introduction

Over the past decade, along with rapid economic growth and social changes, Chinese people have experienced remarkable shifts in its traditional diet and disease patterns [1,2]. As the classic eating pattern shifts, grain intake has significantly decreased; fat intake has dramatically increased; daily intake of salt has been much greater than that recommended; but intake of fruit and vegetables is insufficient [3]. These shifts in the composition of diet have been the basis for the change in nutrient intakes. An imbalanced nutrient intake is associated with increased morbidity and mortality from diseases, including diabetes, hypertension, dyslipidemia, cardiovascular diseases, certain cancers, *etc.* [4,5]. Moreover, assessing intake of nutrient is necessary to monitor nutritional status. It allows us to identify people nutritionally at risk due to inadequate or excessive intake of specific nutrients, to plan and evaluate nutrition intervention projects, and to establish dietary recommendations and food regulations [6,7].

Nowadays, there are predominant types of diets in each country. Some predominant diets are described as healthy diets while others are generally qualified as unhealthy. Both the Mediterranean and Japanese diets are known to be healthy. People in the Mediterranean countries have a low risk of cardiovascular disease, while the Japanese people are famous for their longevity/healthy life expectancy [8]. The Mediterranean diet has long been reported to be the optimal diet for preventing non-communicable diseases and preserving good health [9]. It is characterized by a high intake of cereals, nuts, fruit and vegetables, fish and olive oil, a low intake of dairy products, red meat, processed meats and sweets, and a moderate intake of wine during meals [9,10]. The Japanese diet also covers large amounts of rice, fruit and vegetables, soy-derived proteins and fish, but there is much lower intake of energy and oils/fats [11]. However, the Western diet is characterized by a high consumption of red and processed meats, high-fat dairy products, refined grains, and high-sugar drinks and desserts besides alcoholic beverages, and relatively low intakes of fruit, vegetables, whole-grain foods, fish and poultry [12]. This diet may have negative effects on health, specifically on the risk for obesity, metabolic syndrome, type 2 diabetes, cardiovascular disease as well as cancer [13].

There have been four national nutrition surveys in China, conducted in 1959, 1982, 1992 and 2002. With rapid economic development and urbanization, the pace of nutrition transition has dramatically accelerated in China [2,14]. It is difficult to reflect the nutritional and healthy problems of residents timely for the national nutrition survey carried out once every 10 years. Therefore, the way of national nutrition survey has changed to one cycle every 5 years since 2010 (three years for the first time). Our study was based on the data of the fifth national nutrition survey in south-east China, Zhejiang province, from 2010 to 2012. Although the nutrition survey in Zhejiang province cannot completely reflect the nutritional status all over China, it can indicate the nutritional status in eastern-coast developed provinces and in urbanized provinces. So far, there has been no report which compared the nutrient intakes between Chinese and other types of diets in males and females for different age groups. Therefore, this study aimed to evaluate energy, macro- and micro-nutrients intakes in south-east Chinese diet and compare those between Chinese and Mediterranean (Italian), Japanese and Western (American) diets in males and females for different age groups.

## 2. Methods

### 2.1. Study Design and Subjects

All data of Chinese population in this study were derived from part of the fifth national nutrition survey, China National Nutrition and Health Monitoring Survey, from 2010 to 2012. For Zhejiang province, large cities of Jianggan District in Hangzhou and Jiangdong District in Ningbo were selected in 2010; small-medium cities of Jindong District in Jinhua and Tongxiang County in Jiaxing in 2011; Songyang County in Lishui and Anji County in Huzhou in 2012. We randomly selected six communities in each city, district or county using a randomized probability-proportional-to-size sampling scheme. 25 households in each community constituted a cluster by geography of house address. We randomly selected three clusters, totally 75 households, in each community. Dietary data of 30 households, which were constituted from 25 households in the first cluster and the first five households in second cluster, were collected using three consecutive 24-h dietary recalls. The rest 20 households in second cluster and 25 households in the third cluster were investigated, using instant food questionnaire and food frequency questionnaire to obtain dietary data. The survey was conducted from August to October in each calendar year.

In the present study, we only used dietary data from three consecutive 24-h dietary recalls to compare nutrient intakes between Chinese and other types of diets. That was because 24-h dietary recalls obtained extensive and complete data on all foods and beverages consumed, and then it provided a more precise assessment of intake of nutrients than food frequency methods [15,16]. Overall, 8175 subjects (3908 males and 4267 females) were recruited in this nutrition survey. Among them, 2659 individuals (1274 males and 1385 females) aged 2.0–89.2 years, participating in 24-h dietary recalls, were selected in the present study. The protocol of this survey was approved by the Medical Ethical Committee of Zhejiang Provincial Center of Disease Control and Prevention (CDC), and written informed consent was obtained from all participants.

### 2.2. Dietary Assessment

In this study, dietary intakes at the individual level for all household members aged ≥2 years were collected by well-trained CDC workers, using three consecutive 24-h dietary recalls (two weekdays and one weekend day) [2,17]. For the younger children aged below 12 years, their parents or primary caregivers were asked to recall the child’s food consumption. The investigated information included the type and amount of all food items and consumption place by the aid of food models and pictures. Additionally, home cooking oil and condiment consumption in each household were collected with a household food inventory weighing method on the same 3 days. The percentage of the oil and condiments of each family member was calculated by the ratio of his or her energy intake to the energy intake of all family members [18]. The daily intake of energy and nutrients was averaged over 3 days to assess usual dietary intake. The main nutrients of interest were total energy, carbohydrate, protein, fat, fiber and cholesterol, and intake of vitamins and minerals. Nutrient intakes for each food item consumed were calculated by multiplying the nutrient content listed in the Chinese Food Composition Table (FCT) [19]. Total dietary intake of each nutrient was calculated by adding the intake of that nutrient from each food item consumed.

### 2.3. Anthropometric and Demographic Variables Assessment

Anthropometric measurement (only for subjects aged ≥6 years) and demographic variables were collected from each individual. Body weight and height were respectively measured to the nearest 0.1 kg and 0.1 cm when the subjects wore light clothing and no shoes. Body mass index (BMI) was calculated as weight (kg)/height squared (m^2^). Waist circumference was measured to the nearest 0.1 cm at the midpoint between the top of the iliac crest and the lower margin of the last palpable rib in the mid axillary line using a tape measure. A questionnaire was used to collect information on demographic variables, including age, sex, educational level, marital status, occupation, annual family income, smoking and alcohol consumption and history of diseases.

### 2.4. Data of Mediterranean, Japanese and Western Diets

To compare nutrient intakes between Chinese diet and Mediterranean, Japanese, and Western diets, the data of the third National Food Consumption Survey (INRAN-SCAI 2005–06) in Italy [7], the National Health and Nutrition Survey (2007) in Japan provided by National Institute of Health and Nutrition [20], and the National Health and Nutrition Examination Survey (NHANES 2009–2010) in America from CDC [21] were obtained from corresponding website, respectively.

### 2.5. Statistical Analysis

The data of energy and nutrient intakes were presented as mean ± SD by sex and age. Due to different age categories across countries, age categories were grouped as 1–6 years, 7–14 years, 15–19 years, 20–29 years, 30–39 years, 40–49 years, 50–59 years, 60–69 years, and 70 years and above in data of Japanese and American diets, while age categorized in children (3–9 years), teenagers (10–17 years), younger adults (18–64 years) and older adults (65 years and above) in data of Italian diets. When nutrient intakes were compared between Chinese and other country population, the age categories in Chinese population were grouped according to corresponding age categorization. Food energy was expressed as kilocalories (kcal). Vitamin A was expressed in retinol equivalents (REs). The distributions of variables between male and female subjects were tested using Student’s *t*-test for the continuous variables and Pearson’s χ^2^ test for the categorical variables. Comparison of daily intake of nutrients adjusted for energy by age in Chinese was tested using one-way analysis of variance. Moreover, one-sample *t*-test was used to compare nutrient intakes between Chinese and Japanese, Italian diets, and two-independent-sample *t*-test was used to compare nutrient intakes between Chinese and American diets. All statistical tests were two-tailed. We used the false discovery rate (FDR) to control the false rate of multiple testing [22], and *p* value less than 0.04 was considered statistically significant after FDR adjustment. All analyses were performed using Statistical Analysis System software version 9.2 (SAS Institute Inc., Cary, NC, USA).

## 3. Results

### 3.1. Characteristics of the Study Population in Chinese

The anthropometric and socio-demographic characteristics of the Chinese subjects by sex are presented in Table 1. Of 2659 participants, 47.91% were males. Mean ages of males and females were 49.43 and 47.80 years, respectively. Distributions of BMI, annual family income and history of diseases (hypertension, diabetes, dyslipidemia and stroke) were comparable between males and females. However, males were more likely to be married, higher educational level, longer waist circumference, and more tobacco smoking and alcohol drinking than females. In general, intake of energy and nutrients in males were significantly higher than those in females (Supplementary Table S1). Mean energy intakes were 2194.78 kcal and 1766.14 kcal for males and females, respectively. Compared with females, males consumed more cholesterol (323.10 mg *versus* 289.91 mg), fat (89.06 g *versus* 72.73 g), protein (73.01 g *versus* 61.89 g) and carbohydrate (261.47 g *versus* 219.11 g) except fiber (10.67 g *versus* 10.21 g). However, the mean energy contributions from fat, protein and carbohydrate in males were 36.46%, 13.62% and 48.06%, respectively, which were mildly lower than those in females (37.03%, 14.27% and 49.44%, respectively) with no significant difference in fat. In addition, males consumed more calcium, phosphorus, potassium, sodium, iron, zinc, copper, selenium, manganese, vitamin A, vitamin B_1_, vitamin B_2_ and vitamin E (all *p* < 0.05) except vitamin C (*p* > 0.05). Besides, we also presented the details of energy and nutrient intakes in Chinese by age and sex in Table 2, Table 3, Table 4, Table 5 and Table 6.

**Table 1:** The distributions of socio-demographic characteristics of study subjects by sex in the Chinese group (*N* = 2659).

Variables	Males (*N* = 1274)	Females (*N* = 1385)	*p* value
**Mean ± SD**			
**Age, year**	49.43 ± 18.77	47.80 ± 18.21	**0.0229** *
**Height, cm**	165.15 ± 9.37	154.93 ± 7.22	**<0.0001**
**Weight, kg**	63.37 ± 12.59	55.33 ± 10.17	**<0.0001**
**Body mass index, kg/m^2^**	23.06 ± 3.54	22.95 ± 3.55	0.4271
**Waist circumference, cm**	82.55 ± 11.11	78.65 ± 10.50	**<0.0001**
**N (%)**			
**Education**			**<0.0001**
**Primary school or below**	549 (43.09)	714 (51.55)	
**Junior or senior high school**	636 (49.92)	586 (42.31)	
**College or above**	89 (6.99)	85 (6.14)	
**Missing**	--	--	
**Marital status**			**0.0008**
**No**	205 (16.09)	293 (21.16)	
**Yes**	1069 (83.91)	1092 (78.84)	
**Missing**	--	--	
**Occupation**			**<0.0001**
**Student**	98 (7.69)	113 (8.16)	
**Mental worker**	120 (9.42)	79 (5.70)	
**Manual worker**	553 (43.41)	508 (36.68)	
**Retiree**	203 (15.93)	265 (19.13)	
**Other**	300 (23.55)	420 (30.32)	
**Missing**	--	--	
**Income, yuan/year**			0.7306
**<10,000**	252 (21.25)	291 (22.45)	
**10,000–30,000**	759 (64.00)	811 (62.58)	
**>30,000**	175 (14.76)	194 (14.97)	
**Missing**	88	89	
**Tobacco smoking**			**<0.0001**
**No**	558 (47.57)	1254 (98.51)	
**Yes**	615 (52.43)	19 (1.49)	
**Missing**	101	112	
**Alcohol drinking**			**<0.0001**
**No**	529 (45.1)	991 (77.79)	
**Yes**	644 (54.9)	283 (22.21)	
**Missing**	101	111	
**Hypertension**			0.2011
**No**	899 (78.38)	960 (76.19)	
**Yes**	248 (21.62)	300 (23.81)	
**Missing**	127	125	
**Diabetes**			0.3341
**No**	591 (88.08)	672 (86.38)	
**Yes**	80 (11.92)	106 (13.62)	
**Missing**	603	607	
**Dyslipidemia**			0.8895
**No**	492 (85.86)	582 (85.59)	
**Yes**	81 (14.14)	98 (14.41)	
**Missing**	701	705	
**Stroke**			0.1418
**No**	1153 (98.29)	1262 (98.98)	
**Yes**	20 (1.71)	13 (1.02)	
**Missing**	101	110	

* Bold represents statistical significance.

**Table 2:** Comparison of daily intake of energy and macronutrients by age and sex in the Chinese, Japanese and American groups.

Energy, Macronutrients and Age	Males (*N* = 1274)						Females (*N* = 1385)					
	Chinese		Japanese		American		Chinese		Japanese		American	
	*N*	Mean ± SD	*N*	Mean	*N*	Mean ± SD	*N*	Mean ± SD	*N*	Mean	*N*	Mean ± SD
**Total energy, kcal**												
1–6	37	1287 ± 610	243	1389	552	1522 ± 463 *	33	1027 ± 366	245	1270 *	539	1441 ± 439 *
7–14	63	1777 ± 702	392	2103 *	622	1997 ± 617 *	75	1524 ± 494	403	1871 *	594	1830 ± 551 *
15–19	23	1951 ± 687	201	2440 *	360	2505 ± 923 *	29	1579 ± 568	192	1873 *	316	1806 ± 653
20–29	61	2229 ± 771	304	2183	363	2553 ± 959 *	84	1959 ± 1242	361	1684	446	1877 ± 645
30–39	147	2270 ± 901	540	2208	394	2672 ± 966 *	185	1802 ± 587	661	1725	442	1835 ± 587
40–49	267	2423 ± 842	537	2153 *	407	2601 ± 929 *	292	1898 ± 610	570	1719 *	508	1759 ± 573 *
50–59	259	2339 ± 855	587	2214 *	407	2387 ± 914	301	1858 ± 692	681	1774 *	384	1715 ± 576 *
60–69	255	2150 ± 846	664	2195	399	2071 ± 724	263	1696 ± 765	762	1759	401	1633 ± 563
≥70	162	1982 ± 769	696	1982	417	1884 ± 575	123	1583 ± 589	846	1613	469	1509 ± 515
**Carbohydrate, g**												
1–6	37	161.2 ± 97.9	243	195.9 *	552	207.1 ± 68.4 *	33	115.1 ± 47.0	245	174.7 *	539	195.3 ± 64.1 *
7–14	63	207.9 ± 89.9	392	268.1 *	622	265.9 ± 85.8 *	75	182.3 ± 79.0	403	251.6 *	594	247.3 ± 77.4 *
15–19	23	250.5 ± 102.2	201	333.6 *	360	323.4 ± 125.4 *	29	184.2 ± 78.9	192	245.4 *	316	237.5 ± 90.1 *
20–29	61	254.0 ± 114.1	304	300.2 *	363	319.4 ± 126.4 *	84	238.2 ± 231.3	361	223.2	446	244.6 ± 89.1
30–39	147	269.0 ± 142.3	540	299.3 *	394	322.3 ± 129.7 *	185	212.0 ± 91.8	661	235.4 *	442	232.2 ± 82.0 *
40–49	267	279.8 ± 111.1	537	290.4	407	314.4 ± 120.5 *	292	233.8 ± 91.2	570	233.1	508	226.3 ± 82.8
50–59	259	275.1 ± 115.3	587	296.8 *	407	282.6 ± 114.7	301	234.4 ± 102.9	681	248.2 *	384	217.6 ± 82.8 *
60–69	255	266.0 ± 129.2	664	306.7 *	399	247.9 ± 91.6	263	215.4 ± 116.5	762	256.7 *	401	203.5 ± 74.8
≥70	162	243.6 ± 102.1	696	289.1 *	417	230.5 ± 78.3	123	211.0 ± 91.0	846	244.1 *	469	194.1 ± 70.9
**Protein, g**												
1–6	37	41.8 ± 20.0	243	48.8 *	552	56.6 ± 19.0 *	33	36.7 ± 15.9	245	44.7 *	539	53.9 ± 18.9 *
7–14	63	60.3 ± 26.4	392	75.3 *	622	73.7 ± 26.6 *	75	51.9 ± 17.8	403	67.7 *	594	66.3 ± 22.8 *
15–19	23	69.0 ± 28.9	201	87.5 *	360	96.2 ± 42.2 *	29	54.2 ± 20.5	192	68.3 *	316	64.4 ± 25.3
20–29	61	77.3 ± 27.8	304	76.7	363	99.4 ± 40.4 *	84	69.9 ± 38.3	361	62.9	446	68.7 ± 24.6 *
30–39	147	77.1 ± 32.6	540	76.6	394	104.4 ± 39.1 *	185	63.2 ± 21.6	661	62.5	442	72.0 ± 25.2 *
40–49	267	76.7 ± 29.5	537	75.8	407	102.1 ± 37.9 *	292	63.4 ± 23.3	570	63.1	508	69.0 ± 23.2
50–59	259	76.3 ± 28.7	587	80.5 *	407	95.8 ± 39.0 *	301	66.1 ± 31.6	681	68.1	384	67.1 ± 24.0
60–69	255	73.0 ± 33.4	664	80.9 *	399	84.6 ± 34.1 *	263	62.4 ± 32.5	762	69.1 *	401	65.7 ± 24.9 *
≥70	162	69.0 ± 29.3	696	74.8 *	417	74.4 ± 25.6 *	123	53.9 ± 23.4	846	62.5 *	469	60.0 ± 23.0
**Fat, g**												
1–6	37	53.2 ± 28.2	243	43.8	552	54.0 ± 19.8	33	47.1 ± 21.4	245	42.2	539	51.4 ± 19.2
7–14	63	80.4 ± 45.4	392	69.6	622	73.2 ± 27.9	75	66.9 ± 29.4	403	63.2	594	66.4 ± 25.6
15–19	23	77.1 ± 36.7	201	78.2	360	91.6 ± 40.2	29	71.7 ± 33.1	192	65.4	316	67.7 ± 30.5 *
20–29	61	102.0 ± 44.5	304	66.3 *	363	89.0 ± 40.8 *	84	82.4 ± 41.5	361	55.6 *	446	67.5 ± 29.7 *
30–39	147	95.1 ± 44.3	540	65.1 *	394	96.1 ± 43.4	185	79.3 ± 35.1	661	54.4 *	442	68.1 ± 29.0 *
40–49	267	101.5 ± 51.3	537	60.6 *	407	97.0 ± 45.8	292	80.0 ± 41.2	570	53.9 *	508	63.7 ± 27.6 *
50–59	259	94.8 ± 46.7	587	61.1 *	407	90.2 ± 47.1	301	74.3 ± 36.5	681	52.7 *	384	63.1 ± 27.7
60–69	255	82.1 ± 37.0	664	55.3 *	399	77.3 ± 35.3	263	66.1 ± 33.7	762	47.8 *	401	61.5 ± 29.4
≥70	162	73.3 ± 36.7	696	47.8 *	417	71.3 ± 27.7	123	60.1 ± 34.7	846	40.7 *	469	55.6 ± 23.1 *
**Fiber, g**												
1–6	37	4.7 ± 3.3	243	8.6 *	552	11.5 ± 5.3 *	33	5.2 ± 3.2	245	8.1 *	539	10.8 ± 4.9 *
7–14	63	7.5 ± 5.5	392	13.3 *	622	14.3 ± 6.0 *	75	7.0 ± 4.9	403	12.4 *	594	14.1 ± 6.1 *
15–19	23	8.6 ± 5.5	201	13.2 *	360	16.8 ± 8.1 *	29	7.4 ± 4.0	192	12.1 *	316	12.4 ± 5.8 *
20–29	61	9.8 ± 7.7	304	12.6 *	363	17.9 ± 9.6 *	84	10.0 ± 6.7	361	12.1 *	446	13.9 ± 6.6 *
30–39	147	10.3 ± 6.7	540	12.9 *	394	19.3 ± 9.9 *	185	9.9 ± 6.1	661	12.5 *	442	16.2 ± 8.5 *
40–49	267	10.4 ± 6.3	537	13.2 *	407	19.9 ± 11.4 *	292	10.2 ± 9.2	570	12.5 *	508	15.0 ± 7.7 *
50–59	259	11.5 ± 8.6	587	14.7 *	407	19.3 ± 9.5 *	301	11.9 ± 9.4	681	15.2 *	384	15.9 ± 7.8 *
60–69	255	12.2 ± 8.5	664	16.9 *	399	17.9 ± 9.1 *	263	10.5 ± 6.6	762	16.6 *	401	15.6 ± 7.0 *
≥70	162	11.0 ± 7.9	696	16.5 *	417	17.1 ± 8.7 *	123	10.0 ± 7.5	846	15.3 *	469	15.0 ± 7.2 *
**Cholesterol, mg**												
1–6	37	262 ± 169	243	244	552	185.3 ± 118.1 *	33	297 ± 240	245	234	539	178.1 ± 103.1
7–14	63	308 ± 217	392	352	622	229.8 ± 128.0 *	75	236 ± 160	403	334 *	594	212.8 ± 130.2 *
15–19	23	321 ± 190	201	467 *	360	299.1 ± 181.4	29	373 ± 282	192	382	316	217.2 ± 130.7 *
20–29	61	360 ± 212	304	369	363	330.8 ± 189.8	84	362 ± 247	361	317	446	231.1 ± 142.2 *
30–39	147	353 ± 230	540	365	394	345.5 ± 197.0	185	309 ± 184	661	296	442	240.7 ± 141.3 *
40–49	267	329 ± 256	537	351	407	354.5 ± 206.2	292	295 ± 239	570	311	508	233.4 ± 136.0 *
50–59	259	344 ± 241	587	369	407	343.3 ± 234.1	301	297 ± 229	681	309	384	227.9 ± 136.7 *
60–69	253	315 ± 256	664	353 *	399	304.3 ± 180.5	260	285 ± 234	762	297	401	228.2 ± 143.1
≥70	161	271 ± 183	696	317 *	417	273.4 ± 163.6	122	206 ± 182	846	265 *	469	194.8 ± 111.6 *
***% Total energy from***												
**Carbohydrate, %**												
1–6	37	48.7 ± 13.3	243	58.2 *	552	54.4 ± 7.2 *	33	45.6 ± 10.9	245	56.8 *	539	54.3 ± 7.7 *
7–14	63	47.9 ± 13.1	392	56.1 *	622	53.5 ± 7.3 *	75	47.8 ± 12.4	403	55.2 *	594	54.3 ± 6.9 *
15–19	23	52.0 ± 12.8	201	57.1	360	51.9 ± 8.5	29	46.9 ± 11.9	192	54.5 *	316	52.8 ± 8.1 *
20–29	61	45.6 ± 11.4	304	58.9 *	363	50.5 ± 8.9 *	84	46.7 ± 11.6	361	55.9 *	446	52.5 ± 8.2 *
30–39	147	46.6 ± 12.0	540	59.8 *	394	48.6 ± 10.2	185	46.6 ± 12.3	661	57.4 *	442	51.0 ± 9.3 *
40–49	267	47.0 ± 12.7	537	60.6 *	407	48.8 ± 9.1	292	49.6 ± 12.9	570	57.3 *	508	51.5 ± 9.2
50–59	259	47.7 ± 11.9	587	60.9 *	407	47.9 ± 9.9	301	50.3 ± 11.3	681	58.2 *	384	50.9 ± 9.9
60–69	255	49.3 ± 11.9	664	62.5 *	399	48.5 ± 9.5	263	50.3 ± 12.0	762	60.1 *	401	50.3 ± 9.3
≥70	162	50.1 ± 12.8	696	63.4 *	417	49.3 ± 8.9	123	53.9 ± 14.2	846	62.3 *	469	51.7 ± 8.5 *
**Protein, %**												
1–6	37	13.2 ± 3.2	243	14	552	15.0 ± 3.0 *	33	14.3 ± 3.2	245	14	539	15.1 ± 3.3
7–14	63	13.7 ± 3.3	392	14.4	622	14.9 ± 3.2 *	75	13.8 ± 3.2	403	14.6	594	14.6 ± 3.1
15–19	23	14.1 ± 3.2	201	14.5	360	15.6 ± 4.1	29	14.1 ± 5.0	192	14.7	316	14.6 ± 3.7
20–29	61	14.0 ± 2.9	304	14.2	363	15.8 ± 3.9 *	84	15.0 ± 4.6	361	15	446	14.9 ± 3.5
30–39	147	13.9 ± 3.5	540	14	394	16.1 ± 4.5 *	185	14.5 ± 3.8	661	14.7	442	15.9 ± 3.7 *
40–49	267	13.0 ± 3.5	537	14.3 *	407	16.0 ± 3.7 *	292	13.7 ± 3.9	570	14.8 *	508	16.1 ± 4.0 *
50–59	259	13.4 ± 3.4	587	14.6 *	407	16.3 ± 3.9 *	301	14.3 ± 3.8	681	15.4 *	384	16.0 ± 4.2 *
60–69	255	13.8 ± 3.5	664	14.9 *	399	16.6 ± 4.1 *	263	14.8 ± 4.0	762	15.8 *	401	16.5 ± 4.6 *
≥70	162	14.3 ± 3.9	696	15.1 *	417	16.0 ± 3.8 *	123	13.7 ± 3.4	846	15.4 *	469	16.2 ± 4.2 *
**Fat, %**												
1–6	37	38.2 ± 12.7	243	27.8 *	552	31.8 ± 5.7 *	33	40.6 ± 9.3	245	29.2 *	539	31.8 ± 5.8 *
7–14	63	39.4 ± 12.1	392	29.5 *	622	32.7 ± 5.8 *	75	39.4 ± 11.3	403	30.2 *	594	32.2 ± 5.8 *
15–19	23	35.0 ± 11.4	201	28.4 *	360	32.6 ± 6.6	29	40.3 ± 11.3	192	30.8 *	316	33.3 ± 6.7 *
20–29	61	41.0 ± 11.0	304	26.9 *	363	30.9 ± 6.6 *	84	39.0 ± 10.6	361	29.1 *	446	31.9 ± 6.7 *
30–39	147	38.2 ± 10.8	540	26.2 *	394	32.1 ± 7.7 *	185	39.7 ± 11.0	661	27.9 *	442	32.9 ± 7.3 *
40–49	267	37.2 ± 10.9	537	25.1 *	407	32.9 ± 7.6 *	292	37.4 ± 11.7	570	27.9 *	508	32.1 ± 7.0 *
50–59	259	36.3 ± 10.9	587	24.5 *	407	33.1 ± 7.6 *	301	36.0 ± 10.9	681	26.4 *	384	32.7 ± 7.8 *
60–69	255	34.9 ± 11.0	664	22.6 *	399	33.1 ± 7.6 *	263	35.5 ± 10.9	762	24.1 *	401	33.2 ± 7.9 *
≥70	162	33.2 ± 10.7	696	21.5 *	417	33.7 ± 6.9	123	33.5 ± 12.7	846	22.3 *	469	32.8 ± 6.7

* *p* < 0.04 after FDR adjustment *versus* Chinese.

**Table 3:** Comparison of daily intake of minerals by age and sex in the Chinese, Japanese and American groups.

Minerals and Age	Males (*N* = 1274)						Females (*N* = 1385)					
	Chinese		Japanese		American		Chinese		Japanese		American	
	*N*	Mean ± SD	*N*	Mean	*N*	Mean ± SD	*N*	Mean ± SD	*N*	Mean	*N*	Mean ± SD
**Calcium, mg**												
1–6	37	277 ± 148	243	456 *	552	1044 ± 425 *	33	263 ± 128	245	421 *	539	988 ± 444 *
7–14	63	354 ± 193	392	711 *	622	1134 ± 503 *	75	344 ± 164	403	623 *	594	980 ± 523 *
15–19	23	388 ± 212	201	578 *	360	1233 ± 572 *	29	344 ± 163	192	493 *	316	873 ± 507 *
20–29	61	416 ± 188	304	475 *	363	1189 ± 641 *	84	439 ± 285	361	445	446	902 ± 441 *
30–39	147	468 ± 253	540	451	394	1189 ± 640 *	185	406 ± 190	661	474 *	442	934 ± 421 *
40–49	267	453 ± 226	537	472	407	1107 ± 568 *	292	389 ± 213	570	466 *	508	865 ± 403 *
50–59	259	466 ± 248	587	517 *	407	1053 ± 565 *	301	433 ± 264	681	542 *	384	868 ± 431 *
60–69	255	458 ± 241	664	589 *	399	940 ± 558 *	263	430 ± 256	762	580 *	401	815 ± 433 *
≥70	162	473 ± 267	696	587 *	417	880 ± 429 *	123	388 ± 236	846	553 *	469	806 ± 412 *
**Phosphorus, mg**												
1–6	37	595 ± 282	243	744 *	552	1135 ± 370 *	33	511 ± 198	245	683 *	539	1071 ± 376 *
7–14	63	812 ± 328	392	1140 *	622	1364 ± 484 *	75	707 ± 214	403	1033 *	594	1208 ± 405 *
15–19	23	925 ± 364	201	1192 *	360	1613 ± 627 *	29	739 ± 269	192	955 *	316	1132 ± 475 *
20–29	61	1025 ± 347	304	1030	363	1640 ± 655 *	84	940 ± 532	361	875	446	1192 ± 436 *
30–39	147	1054 ± 440	540	1042	394	1740 ± 658 *	185	862 ± 274	661	895	442	1257 ± 441 *
40–49	267	1040 ± 373	537	1046	407	1683 ± 624 *	292	864 ± 300	570	898	508	1181 ± 403 *
50–59	259	1045 ± 379	587	1115 *	407	1567 ± 618 *	301	915 ± 413	681	984 *	384	1157 ± 418 *
60–69	255	1014 ± 438	664	1158 *	399	1396 ± 564 *	263	874 ± 433	762	1010 *	401	1128 ± 423 *
≥70	162	969 ± 422	696	1078 *	417	1261 ± 434 *	123	773 ± 329	846	925 *	469	1052 ± 398 *
**Potassium, mg**												
1–6	37	950 ± 465	243	1571 *	552	2108 ± 670 *	33	924 ± 449	245	1455 *	539	1995 ± 649 *
7–14	63	1375 ± 746	392	2356 *	622	2272 ± 797 *	75	1231 ± 470	403	2145 *	594	2134 ± 773 *
15–19	23	1661 ± 878	201	2329 *	360	2761 ± 1177 *	29	1389 ± 639	192	2052 *	316	1968 ± 782 *
20–29	61	1704 ± 763	304	2181 *	363	2860 ± 1195 *	84	1672 ± 998	361	1913 *	446	2195 ± 863 *
30–39	147	1808 ± 785	540	2204 *	394	3132 ± 1216 *	185	1577 ± 614	661	2018 *	442	2431 ± 890 *
40–49	267	1771 ± 715	537	2266 *	407	3180 ± 1343 *	292	1564 ± 704	570	2088 *	508	2392 ± 851 *
50–59	259	1827 ± 841	587	2518 *	407	3082 ± 1164 *	301	1693 ± 846	681	2425 *	384	2450 ± 888 *
60–69	255	1800 ± 850	664	2742 *	399	2906 ± 1133 *	263	1603 ± 834	762	2613 *	401	2390 ± 891 *
≥70	162	1733 ± 911	696	2650 *	417	2731 ± 967 *	123	1463 ± 789	846	2401 *	469	2323 ± 857 *
**Sodium, mg**												
1–6	37	3719 ± 3757	243	2559	552	2242 ± 816 *	33	2749 ± 1813	245	2323	539	2146 ± 788
7–14	63	4374 ± 2242	392	3858	622	3247 ± 1212 *	75	4371 ± 2501	403	3543 *	594	2953 ± 1004 *
15–19	23	4228 ± 1807	201	4606	360	4135 ± 1689	29	3637 ± 2015	192	3740	316	2932 ± 1195
20–29	61	4711 ± 2125	304	4488	363	4248 ± 1729	84	5112 ± 3326	361	3701 *	446	3116 ± 1107 *
30–39	147	6081 ± 4251	540	4449 *	394	4428 ± 1713 *	185	5112 ± 3393	661	3776 *	442	3061 ± 1063 *
40–49	267	6664 ± 6882	537	4606 *	407	4346 ± 1768 *	292	6121 ± 10810	570	3898 *	508	2905 ± 1032 *
50–59	259	7344 ± 6507	587	4961 *	407	3955 ± 1636 *	301	5703 ± 4413	681	4252 *	384	2791 ± 1037 *
60–69	255	6061 ± 5731	664	4961 *	399	3575 ± 1404 *	263	5307 ± 5011	762	4291 *	401	2775 ± 1071 *
≥70	162	5540 ± 3385	696	4685 *	417	3210 ± 1061 *	123	5148 ± 4228	846	4094 *	469	2553 ± 968 *
**Magnesium, mg**												
1–6	37	148 ± 72	243	158	552	209 ± 69 *	33	141 ± 61	245	144	539	196 ± 65 *
7–14	63	212 ± 101	392	238	622	244 ± 89 *	75	198 ± 66	403	216 *	594	227 ± 81 *
15–19	23	241 ± 96	201	248	360	300 ± 122 *	29	202 ± 68	192	216	316	217 ± 84
20–29	61	276 ± 110	304	241 *	363	326 ± 137 *	84	261 ± 168	361	204 *	446	245 ± 95
30–39	147	295 ± 131	540	250 *	394	358 ± 139 *	185	248 ± 88	661	219 *	442	272 ± 106 *
40–49	267	299 ± 113	537	257 *	407	357 ± 149 *	292	254 ± 111	570	224 *	508	265 ± 100
50–59	259	299 ± 116	587	279 *	407	336 ± 127 *	301	270 ± 123	681	256	384	271 ± 102
60–69	255	300 ± 127	664	301	399	309 ± 125	263	262 ± 130	762	274	401	258 ± 95
≥70	162	284 ± 127	696	284	417	283 ± 125	123	231 ± 99	846	248	469	241 ± 94
**Iron, mg**												
1–6	37	11.3 ± 5.1	243	4.9 *	552	11.7 ± 5.1	33	11.3 ± 6.7	245	4.5 *	539	11.1 ± 5.1
7–14	63	17.3 ± 8.9	392	7.3 *	622	15.5 ± 6.8	75	14.6 ± 5.7	403	6.6 *	594	14.2 ± 6.1
15–19	23	18.0 ± 7.6	201	8.4 *	360	18.5 ± 9.6	29	15.0 ± 5.5	192	7.2 *	316	12.6 ± 6.1
20–29	61	21.5 ± 8.8	304	7.8 *	363	18.0 ± 9.2 *	84	18.7 ± 11.1	361	7.1 *	446	14.0 ± 7.2 *
30–39	147	22.5 ± 10.5	540	8 *	394	18.8 ± 9.2 *	185	18.9 ± 7.0	661	7.5 *	442	14.0 ± 6.1 *
40–49	267	23.1 ± 9.7	537	8 *	407	18.5 ± 8.2 *	292	19.9 ± 18.8	570	7.1 *	508	12.7 ± 5.3 *
50–59	259	23.7 ± 11.8	587	8.7 *	407	17.0 ± 8.4 *	301	21.4 ± 17.3	681	8.3 *	384	12.9 ± 6.1 *
60–69	255	22.9 ± 12.3	664	9.4 *	399	15.8 ± 8.1 *	263	19.9 ± 12.0	762	9 *	401	12.3 ± 5.8 *
≥70	162	20.4 ± 9.0	696	9 *	417	16.0 ± 7.3 *	123	17.5 ± 12.1	846	8.1 *	469	12.4 ± 6.0 *
**Zinc, mg**												
1–6	37	6.3 ± 3.0	243	5.8	552	8.7 ± 3.5 *	33	5.5 ± 2.4	245	5.4	539	8.3 ± 3.5 *
7–14	63	9.2 ± 3.6	392	9.3	622	11.5 ± 6.3 *	75	7.8 ± 2.3	403	8.2	594	9.9 ± 4.2 *
15–19	23	10.6 ± 3.9	201	11	360	13.9 ± 12.5 *	29	8.1 ± 2.7	192	8.2	316	8.9 ± 4.3
20–29	61	11.5 ± 3.8	304	9.4 *	363	13.7 ± 6.7 *	84	10.5 ± 7.2	361	7.5 *	446	9.6 ± 4.6
30–39	147	12.0 ± 5.0	540	9.4 *	394	14.6 ± 6.2 *	185	9.6 ± 3.0	661	7.4 *	442	10.2 ± 6.4
40–49	267	12.4 ± 4.4	537	9.1 *	407	14.3 ± 6.2 *	292	10.0 ± 3.5	570	7.3 *	508	9.7 ± 5.0
50–59	259	12.1 ± 4.3	587	9.2 *	407	13.6 ± 8.3 *	301	10.3 ± 4.7	681	7.7 *	384	9.9 ± 7.2
60–69	255	11.6 ± 5.2	664	9.3 *	399	11.9 ± 6.1	263	9.7 ± 5.0	762	7.8 *	401	9.2 ± 4.5
≥70	162	10.6 ± 4.0	696	8.6 *	417	11.7 ± 6.1 *	123	8.4 ± 3.3	846	7.2 *	469	9.2 ± 4.9
**Selenium, ug**												
1–6	37	26.9 ± 14.2	243	–––	552	74.5 ± 26.9 *	33	27.4 ± 17.7	245	–––	539	71.6 ± 26.3 *
7–14	63	42.8 ± 31.4	392	–––	622	101.5 ± 39.2 *	75	31.8 ± 14.3	403	–––	594	92.1 ± 34.8 *
15–19	23	41.8 ± 22.0	201	–––	360	130.1 ± 59.6 *	29	36.4 ± 21.7	192	–––	316	87.6 ± 35.2 *
20–29	61	50.8 ± 24.9	304	–––	363	138.5 ± 62.1 *	84	48.7 ± 37.7	361	–––	446	95.8 ± 35.8 *
30–39	147	51.4 ± 28.7	540	–––	394	142.7 ± 58.2 *	185	44.8 ± 23.9	661	–––	442	99.6 ± 37.6 *
40–49	267	50.7 ± 33.3	537	–––	407	140.4 ± 54.1 *	292	39.4 ± 21.8	570	–––	508	94.6 ± 36.3 *
50–59	259	52.1 ± 29.7	587	–––	407	129.0 ± 56.4 *	301	45.8 ± 37.0	681	–––	384	91.0 ± 35.3 *
60–69	255	48.6 ± 32.6	664	–––	399	117.2 ± 47.7 *	263	46.3 ± 34.9	762	–––	401	91.1 ± 38.0 *
≥70	162	46.0 ± 26.7	696	–––	417	103.0 ± 37.1 *	123	35.9 ± 23.9	846	–––	469	81.3 ± 34.5 *
**Copper, mg**												
1–6	37	1.2 ± 0.9	243	0.75 *	552	0.8 ± 0.3 *	33	1.0 ± 0.6	245	0.67 *	539	0.8 ± 0.4
7–14	63	2.0 ± 1.4	392	1.13 *	622	1.0 ± 0.5 *	75	1.4 ± 0.5	403	1.03 *	594	1.0 ± 0.4 *
15–19	23	1.9 ± 1.1	201	1.32 *	360	1.3 ± 0.9 *	29	1.6 ± 1.0	192	1.03 *	316	0.9 ± 0.4 *
20–29	61	2.2 ± 1.4	304	1.23 *	363	1.4 ± 0.7 *	84	2.0 ± 1.5	361	1.00 *	446	1.1 ± 0.6 *
30–39	147	2.1 ± 1.2	540	1.24 *	394	1.5 ± 0.7 *	185	1.8 ± 0.8	661	1.02 *	442	1.2 ± 0.7 *
40–49	267	2.2 ± 1.0	537	1.25 *	407	1.5 ± 0.7 *	292	1.9 ± 1.0	570	1.02 *	508	1.2 ± 0.9 *
50–59	259	2.2 ± 1.2	587	1.32 *	407	1.4 ± 0.7 *	301	2.1 ± 1.6	681	1.16 *	384	1.2 ± 0.8 *
60–69	255	2.2 ± 1.2	664	1.4 *	399	1.3 ± 0.6 *	263	1.8 ± 1.2	762	1.23 *	401	1.2 ± 1.1 *
≥70	162	2.0 ± 1.2	696	1.33 *	417	1.3 ± 1.5 *	123	1.8 ± 1.1	846	1.15 *	469	1.1 ± 0.7 *

* *p* < 0.04 after FDR adjustment, *versus* Chinese.

**Table 4:** Comparison of daily intake of vitamins by age and sex in the Chinese, Japanese and American groups.

Vitamins and Age	Males (*N* = 1274)						Females (*N* = 1385)					
	Chinese		Japanese		American		Chinese		Japanese		American	
	*N*	Mean ± SD	*N*	Mean	*N*	Mean ± SD	*N*	Mean ± SD	*N*	Mean	*N*	Mean ± SD
**Vitamin A, μg REs ^&^**												
1–6	37	277 ± 397	243	419 *	552	589 ± 263 *	33	282 ± 236	245	407 *	539	570 ± 288 *
7–14	63	377 ± 395	392	618 *	622	641 ± 369 *	75	270 ± 184	403	606 *	594	569 ± 307 *
15–19	23	364 ± 237	201	805 *	360	640 ± 390 *	29	398 ± 264	192	561 *	316	469 ± 338
20–29	61	482 ± 630	304	603	363	617 ± 469	84	400 ± 335	361	627 *	446	550 ± 463 *
30–39	147	577 ± 1020	540	592	394	669 ± 522	185	415 ± 345	661	529 *	442	584 ± 398 *
40–49	267	444 ± 498	537	630 *	407	650 ± 461 *	292	422 ± 434	570	519 *	508	569 ± 569 *
50–59	259	554 ± 969	587	601	407	650 ± 451	301	467 ± 559	681	612 *	384	635 ± 527 *
60–69	255	453 ± 466	664	674 *	399	628 ± 463 *	263	466 ± 549	762	695 *	401	620 ± 634 *
≥70	162	445 ± 444	696	750 *	417	772 ± 949 *	123	361 ± 332	846	617 *	469	651 ± 468 *
**Vitamin B_1_, mg**												
1–6	37	0.51 ± 0.28	243	0.61 *	552	1.30 ± 0.52 *	33	0.44 ± 0.17	245	0.58 *	539	1.22 ± 0.46 *
7–14	63	0.72 ± 0.38	392	1.28 *	622	1.72 ± 0.68 *	75	0.62 ± 0.24	403	1.19 *	594	1.54 ± 0.60 *
15–19	23	0.80 ± 0.31	201	1.42 *	360	1.97 ± 0.90 *	29	0.64 ± 0.26	192	0.95 *	316	1.33 ± 0.58 *
20–29	61	0.98 ± 0.47	304	1.39 *	363	1.97 ± 0.97 *	84	0.82 ± 0.49	361	1.05 *	446	1.48 ± 0.67 *
30–39	147	1.03 ± 0.52	540	1.23 *	394	2.05 ± 1.06 *	185	0.74 ± 0.29	661	1.19 *	442	1.48 ± 0.61 *
40–49	267	1.14 ± 0.64	537	1.25 *	407	2.01 ± 0.90 *	292	0.80 ± 0.35	570	1.27 *	508	1.35 ± 0.54 *
50–59	259	1.08 ± 0.61	587	1.42 *	407	1.84 ± 0.83 *	301	0.82 ± 0.42	681	1.48 *	384	1.36 ± 0.58 *
60–69	255	0.91 ± 0.46	664	1.44 *	399	1.70 ± 0.81 *	263	0.74 ± 0.41	762	1.88 *	401	1.31 ± 0.57 *
≥70	162	0.87 ± 0.48	696	1.71 *	417	1.65 ± 0.68 *	123	0.65 ± 0.31	846	2.3 *	469	1.31 ± 0.57 *
**Vitamin B_2_, mg**												
1–6	37	0.55 ± 0.29	243	0.91 *	552	1.91 ± 0.68 *	33	0.48 ± 0.24	245	0.82 *	539	1.81 ± 0.69 *
7–14	63	0.71 ± 0.33	392	1.38 *	622	2.14 ± 0.84 *	75	0.59 ± 0.21	403	1.33 *	594	1.85 ± 0.71 *
15–19	23	0.81 ± 0.39	201	1.75 *	360	2.32 ± 1.17 *	29	0.66 ± 0.25	192	1.27 *	316	1.59 ± 0.84 *
20–29	61	0.85 ± 0.36	304	1.43 *	363	2.33 ± 1.42 *	84	0.81 ± 0.44	361	1.39 *	446	1.77 ± 1.01 *
30–39	147	0.91 ± 0.50	540	1.34 *	394	2.54 ± 1.37 *	185	0.73 ± 0.29	661	1.41 *	442	1.87 ± 0.80 *
40–49	267	0.90 ± 0.46	537	1.39 *	407	2.44 ± 1.13 *	292	0.73 ± 0.35	570	1.28 *	508	1.79 ± 0.80 *
50–59	259	0.89 ± 0.40	587	1.62 *	407	2.37 ± 1.23 *	301	0.78 ± 0.44	681	1.56 *	384	1.83 ± 0.83 *
60–69	255	0.85 ± 0.46	664	1.57 *	399	2.16 ± 1.11 *	263	0.75 ± 0.48	762	1.57 *	401	1.75 ± 0.84 *
≥70	162	0.78 ± 0.37	696	1.5 *	417	2.17 ± 0.91 *	123	0.61 ± 0.31	846	1.76 *	469	1.74 ± 0.77 *
**Niacin, mg**												
1–6	37	8.2 ± 4.0	243	8.3	552	16.2 ± 6.5 *	33	7.3 ± 3.6	245	7.6	539	15.2 ± 6.2 *
7–14	63	12.9 ± 5.8	392	12.9	622	23.1 ± 8.9 *	75	10.4 ± 3.6	403	11.5 *	594	20.7 ± 7.8 *
15–19	23	15.8 ± 7.6	201	16.5	360	30.2 ± 14.7 *	29	11.6 ± 5.2	192	13.3	316	19.7 ± 8.3 *
20–29	61	17.1 ± 6.0	304	16.9	363	31.9 ± 15.5 *	84	14.7 ± 9.1	361	13.2	446	22.2 ± 9.5 *
30–39	147	17.5 ± 8.3	540	17.6	394	33.4 ± 14.0 *	185	13.5 ± 4.7	661	13.3	442	22.0 ± 8.2 *
40–49	267	18.3 ± 8.0	537	17.5	407	31.8 ± 13.3 *	292	13.9 ± 5.6	570	14.1	508	20.8 ± 8.1 *
50–59	259	18.1 ± 8.0	587	18.9	407	29.0 ± 13.1 *	301	14.6 ± 7.6	681	15.2	384	20.5 ± 8.3 *
60–69	255	16.6 ± 7.6	664	18.7 *	399	25.7 ± 11.1 *	263	13.9 ± 7.5	762	15.4 *	401	19.6 ± 8.1 *
≥70	162	15.0 ± 7.2	696	16.2 *	417	24.4 ± 10.5 *	123	11.0 ± 4.5	846	13.5 *	469	18.5 ± 8.1 *
**Vitamin C, mg**												
1–6	37	24.8 ± 20.5	243	55 *	552	83 ± 55 *	33	32.8 ± 22.6	245	55 *	539	83 ± 56 *
7–14	63	44.7 ± 45.6	392	83 *	622	77 ± 58 *	75	43.0 ± 37.1	403	79 *	594	82 ± 80 *
15–19	23	109.9 ± 156.0	201	108	360	101 ± 92	29	57.2 ± 44.3	192	91 *	316	79 ± 69 *
20–29	61	62.4 ± 143.2	304	92	363	92 ± 96	84	58.9 ± 40.3	361	105 *	446	84 ± 86 *
30–39	147	66.7 ± 45.2	540	85 *	394	91 ± 79 *	185	62.3 ± 40.9	661	95 *	442	78 ± 67 *
40–49	266	62.1 ± 43.2	537	87 *	407	94 ± 92 *	292	60.5 ± 40.5	570	112 *	508	81 ± 71 *
50–59	259	61.5 ± 44.0	587	115 *	407	93 ± 86 *	301	67.9 ± 50.7	681	144 *	384	85 ± 64 *
60–69	255	68.5 ± 49.3	664	135 *	399	90 ± 88 *	263	72.9 ± 60.5	762	159 *	401	77 ± 60
≥70	162	71.6 ± 52.1	696	131 *	417	89 ± 74 *	123	59.8 ± 40.7	846	142 *	469	82 ± 56 *
**Vitamin E, mg**												
1–6	37	17 ± 11	243	4.9 *	552	4.8 ± 2.6 *	33	17 ± 9	245	4.5 *	539	4.3 ± 2.1 *
7–14	63	23 ± 17	392	6.7 *	622	6.1 ± 3.1 *	75	24 ± 15	403	6.3 *	594	6.2 ± 3.9 *
15–19	23	22 ± 11	201	9.3 *	360	7.7 ± 4.5 *	29	26 ± 15	192	6.9 *	316	5.8 ± 3.0 *
20–29	61	35 ± 20	304	7.9 *	363	8.1 ± 5.6 *	84	30 ± 19	361	6.5 *	446	6.3 ± 3.5 *
30–39	147	32 ± 18	540	7.4 *	394	8.7 ± 4.9 *	185	29 ± 17	661	8.3 *	442	6.9 ± 5.3 *
40–49	267	35 ± 24	537	8.1 *	407	8.7 ± 5.5 *	292	29 ± 17	570	8.9 *	508	6.6 ± 4.3 *
50–59	259	33 ± 22	587	9.5 *	407	8.1 ± 5.1 *	301	27 ± 15	681	8.9 *	384	6.9 ± 4.4 *
60–69	255	30 ± 17	664	9.1 *	399	7.4 ± 4.3 *	263	25 ± 13	762	9.8 *	401	6.7 ± 4.4 *
≥70	162	27 ± 16	696	9.9 *	417	7.7 ± 4.9 *	123	24 ± 14	846	11.8 *	469	6.2 ± 3.7 *

^&^ REs: Retinol equivalents; * *p* < 0.04 after FDR adjustment, *versus* Chinese.

**Table 5:** Comparison of daily intake of energy and nutrients ^#^ by age in children ^ and males in the Chinese and Italian groups.

Energy and Nutrients	Children (3–9 Years)		Males (10–17 Years)		Males (18–64 Years)		Males (65 Years and Above)	
	Chinese (*N* = 112)	Italian (*N* = 193)	Chinese (*N* = 53)	Italian (*N* = 108)	Chinese (*N* = 896)	Italian (*N* = 1068)	Chinese (*N* = 263)	Italian (*N* = 202)
**Total energy, kcal**	1322.2 ± 560.9	1914 ± 488 *	1889 ± 684	2576 ± 744 *	2330 ± 865	2390 ± 650 *	1981 ± 740	2296 ± 556 *
**Carbohydrate, g**	157.4 ± 81.0	239.8 ± 69.8 *	231.5 ± 92.8	326.7 ± 110.2 *	274.9 ± 123.8	283.1 ± 88.7	243.6 ± 101.4	274.9 ± 81.4 *
**Protein, g**	44.9 ± 20.2	74.1 ± 18.5 *	63.3 ± 26.1	99.3 ± 26.2 *	76.9 ± 31.0	92.6 ± 25.3 *	67.9 ± 28.1	88.2 ± 21.4 *
**Fat, g**	57.9 ± 30.3	79.5 ± 22.8 *	81.5 ± 45.5	105.4 ± 32.3 *	95.9 ± 46.4	95.4 ± 29.5	73.9 ± 35.3	87.0 ± 23.4 *
**Fiber, g**	5.5 ± 3.7	14.4 ± 5.2 *	8.6 ± 5.8	18.1 ± 5.9 *	11.0 ± 7.6	19.6 ± 7.3 *	11.3 ± 8.1	21.6 ± 8.2 *
**Cholesterol, mg**	275.1 ± 198.4	286 ± 118	301 ± 217	355 ± 153	339 ± 247	331 ± 157	281 ± 202	302 ± 137
***% Total energy from***								
**Carbohydrate, %**	47.5 ± 12.3	46.8 ± 5.8	50.3 ± 13.6	47.2 ± 5.6	47.4 ± 12.0	44.3 ± 6.2 *	49.8 ± 12.8	44.8 ± 6.3 *
**Protein, %**	13.8 ± 3.3	15.7 ± 2.3 *	13.5 ± 3.0	15.6 ± 1.9 *	13.5 ± 3.4	15.7 ± 2.2 *	14.1 ± 3.8	15.5 ± 2 *
**Fat, %**	39.2 ± 11.1	37.4 ± 4.9	37.6 ± 12.7	36.9 ± 4.9	37.0 ± 10.9	36.0 ± 5.3 *	33.8 ± 11.1	34.3 ± 5.7
***Minerals***								
**Calcium, mg**	292.1 ± 140.0	749 ± 252 *	381 ± 216	892 ± 344 *	460 ± 241	799 ± 337 *	458 ± 246	825 ± 331 *
**Phosphorus, mg**	619.1 ± 254.6	1180 ± 299 *	875 ± 339	1479 ± 396 *	1049 ± 406	1386 ± 389 *	951 ± 387	1331 ± 332 *
**Potassium, mg**	1050.5 ± 473.0	2441 ± 633 *	1552 ± 883	3123 ± 879 *	1811 ± 808	3218 ± 921 *	1702 ± 824	3300 ± 859 *
**Magnesium, mg**	162.7 ± 70.4	230 ± 69 *	229 ± 99	286 ± 75 *	300 ± 122	305 ± 93	282 ± 119	295 ± 81
**Zinc, mg**	6.7 ± 2.7	9.9 ± 2.9 *	9.9 ± 3.8	13.3 ± 3.9 *	12.2 ± 4.7	12.6 ± 3.9 *	10.5 ± 3.8	12.2 ± 3.2 *
***Vitamins***								
**Vitamin A, ug REs ^&^**	299.7 ± 346.8	740 ± 941 *	352 ± 316	802 ± 767 *	507 ± 770	890 ± 1004 *	429 ± 407	888 ± 851 *
**Vitamin B_1_, mg**	0.5 ± 0.2	0.92 ± 0.3 *	0.78 ± 0.40	1.23 ± 0.46 *	1.06 ± 0.58	1.11 ± 0.38 *	0.85 ± 0.45	1.01 ± 0.3 *
**Vitamin B_2_, mg**	0.6 ± 0.3	1.43 ± 0.41 *	0.76 ± 0.38	1.69 ± 0.53 *	0.89 ± 0.45	1.53 ± 0.5 *	0.78 ± 0.35	1.48 ± 0.41 *
**Vitamin C, mg**	31.5 ± 27.4	107 ± 64 *	77 ± 114	136 ± 93 *	64 ± 57	126 ± 79 *	71 ± 50	127 ± 74 *
**Vitamin E, mg**	18.6 ± 10.7	10.4 ± 3.5 *	24.2 ± 17.7	13.9 ± 5 *	33.3 ± 21.1	13.5 ± 4.6 *	26.9 ± 15.6	13.3 ± 4.5 *

^#^ Mean ± SD; ^&^ REs: Retinol equivalents; ^ Two infants (< 3 years) were not included, with males and females grouped in the case of children; * *p* < 0.04 after FDR adjustment, *versus* Chinese.

**Table 6:** Comparison of intake of energy and nutrients ^#^ by age in females in the Chinese and Italian groups.

Energy and Nutrients	Females (10–17 Years)		Females (18–64 Years)		Females (65 Years and above)	
	Chinese (*N* = 75)	Italian (*N* = 139)	Chinese (*N* = 1027)	Italian (*N* = 1245)	Chinese (*N* = 231)	Italian (*N* = 316)
**Total energy, kcal**	1549 ± 516	2091 ± 532 *	1845 ± 750	1939 ± 526 *	1613 ± 558	1834 ± 486 *
**Carbohydrate, g**	181.8 ± 82.5	263.1 ± 80.1 *	227.8 ± 118.8	236.5 ± 75.3 *	210.2 ± 89.3	233.7 ± 71.7 *
**Protein, g**	53.1 ± 17.9	81.8 ± 20.1 *	64.5 ± 29.6	76 ± 19.5 *	57.8 ± 23.5	71.4 ± 18.8 *
**Fat, g**	69.2 ± 30.2	86 ± 23.1 *	76.5 ± 38.5	79.1 ± 23.4 *	61.7 ± 31.2	69.6 ± 22.2 *
**Fiber, g**	6.8 ± 4.6	16.4 ± 5.8 *	10.7 ± 8.3	17.7 ± 6.3 *	10.0 ± 6.7	18.7 ± 6.7 *
**Cholesterol, mg**	283 ± 205	311 ± 144	304 ± 230	265 ± 125 *	239 ± 198	243 ± 106
***% Total energy from***						
**Carbohydrate, %**	46.6 ± 12.3	46.8 ± 5.8	49.1 ± 12.3	45.5 ± 6.3 *	52.2 ± 13.0	47.8 ± 7.3
**Protein, %**	14.1 ± 4.0	15.8 ± 2.2 *	14.2 ± 3.9	15.9 ± 2.3 *	14.5 ± 3.9	15.7 ± 2.4 *
**Fat, %**	40.2 ± 11.5	37.2 ± 5 *	37.3 ± 11.3	36.8 ± 5.3	34.2 ± 11.7	34.1 ± 6.1
***Minerals***						
**Calcium, mg**	339 ± 166	770 ± 280 *	415 ± 241	730 ± 277 *	409 ± 236	754 ± 290 *
**Phosphorus, mg**	712 ± 214	1252 ± 333 *	886 ± 391	1168 ± 312 *	818 ± 317	1117 ± 305 *
**Potassium, mg**	1241 ± 494	2737 ± 796 *	1621 ± 800	2861 ± 797 *	1515 ± 733	2822 ± 794 *
**Magnesium, mg**	199 ± 67	251 ± 91	259 ± 122	257 ± 74	245 ± 101	243 ± 66
**Zinc, mg**	7.9 ± 2.3	10.9 ± 3 *	10.0 ± 4.6	10.6 ± 3 *	8.9 ± 3.2	9.9 ± 2.9 *
***Vitamins***						
**Vitamin A, ug REs ^&^**	318 ± 221	751 ± 855 *	437 ± 471	818 ± 885 *	418 ± 450	773 ± 466 *
**Vitamin B_1_, mg**	0.64 ± 0.24	1 ± 0.32 *	0.79 ± 0.39	0.95 ± 0.32 *	0.68 ± 0.32	0.86 ± 0.26 *
**Vitamin B_2_, mg**	0.61 ± 0.22	1.42 ± 0.4 *	0.76 ± 0.42	1.38 ± 0.43 *	0.66 ± 0.33	1.31 ± 0.39 *
**Vitamin C, mg**	48 ± 40	128 ± 92 *	64 ± 47	123 ± 74 *	67 ± 52	127 ± 84 *
**Vitamin E, mg**	25.4 ± 16.2	11.8 ± 3.5 *	27.8 ± 16.3	11.9 ± 3.8 *	23.6 ± 12.7	10.9 ± 3.7 *

^#^ Mean ± SD; ^&^ REs: Retinol equivalents; * *p* < 0.04 after FDR adjustment, *versus* Chinese.

### 3.2. Comparison of Age- and Sex-Specific Intake of Energy and Macronutrients between Chinese and Italian, Japanese and American Subjects

Table 2, Table 5 and Table 6 show the comparison of intake of energy and macronutrients by age and sex. Compared with the subjects of other three countries, Chinese subjects consumed much less fiber in all age groups of both sexes (all *p* < 0.04). In general, compared with the Japanese subjects, young Chinese subjects consumed lower total energy, while adult Chinese subjects consumed higher total energy; compared with the American subjects, Chinese males and young Chinese females consumed lower total energy, while adult Chinese females consumed higher total energy. Carbohydrate and protein intakes in Chinese subjects were lower than those in Japanese and American subjects. Fat intake in Chinese subjects was higher than that in Japanese adults of both sexes and American females, while similar to that in American males (except 20–29 age group). In addition, Chinese, Japanese, American and Italian subjects consumed similar cholesterol, except American females. Furthermore, total energy and macronutrients in Chinese subjects were lower than those in Italian subjects in both sexes except for a similar intake of cholesterol.

The percent energy from carbohydrate in Chinese subjects ranged from 45.6% to 53.9%. It was lower than that in Japanese and American subjects, while higher than that in adult Italian subjects. However, the energy contribution from fat in Chinese subjects ranged between 33.2% and 41.0%. It was higher than that in Japanese and American subjects in both sexes (except ≥70 age group), while similar to that in Italian subjects. The contribution of protein to energy in Chinese subjects (13.0%–15.0%) was lower than that in Japanese, American and Italian subjects; nevertheless, there were no significant differences for Japanese subjects aged <40 years and American females aged <30 years.

### 3.3. Comparison of Age- and Sex-Specific Intake of Minerals between Chinese and Italian, Japanese and American Subjects

For macro-minerals, daily intakes of calcium, phosphorus and potassium in Chinese subjects were generally lower than those in Japanese, American and Italian subjects (Table 3, Table 5 and Table 6). Nevertheless, Chinese subjects consumed a higher intake of sodium than Japanese and American subjects (Table 3). In addition, magnesium intake in Chinese subjects was higher than that in younger adult Japanese subjects, but lower than that in American males aged <60 years and females aged 1–14 and 30–39 years, and Italian children and males aged below 18 years.

For micro-minerals, intakes of iron and copper in both male and female age groups in Chinese subjects were higher than those in Japanese and American subjects, but selenium intake was lower than that in American subjects (Table 3). Zinc intake in Chinese subjects was higher than that in adult Japanese subjects, but lower than that in American males and Italian subjects (Table 3, Table 5 and Table 6).

### 3.4. Comparison of Age- and Sex-Specific Intake of Vitamins between Chinese and Italian, Japanese and American Subjects

For vitamins, intakes of vitamin B_1_ and vitamin B_2_ were lower among Chinese subjects in all age groups of both sexes, compared with those in the people of the other three countries; but vitamin E intake was higher (all *p* < 0.04, Table 4, Table 5 and Table 6). Compared with the people of other three countries, Chinese subjects consumed a lower level of vitamin A and vitamin C in both sexes (Table 4, Table 5 and Table 6), with no significant differences across several age groups in Japanese and American subjects (Table 4). Although niacin intake by age and sex in Chinese subjects was lower than that in American subjects (all *p* < 0.04), these significant differences were only found in Japanese older adults and females aged 7–14 years (Table 4).

### 3.5. Comparison of Age- and Sex-Specific Intake of Nutrients Adjusted for Energy between Chinese and Italian Subjects

As presented in Supplementary Tables S2 and S3, intakes of fiber, calcium, phosphorus, potassium, zinc, vitamin A, vitamin B_1_, vitamin B_2_ and vitamin C after energy adjusted in Chinese subjects were lower than those in Italian subjects, while intakes of cholesterol, iron, magnesium and vitamin E were higher among Chinese subjects relative to Italian subjects (*p* < 0.04). These results were quite similar before and after energy adjustment.

## 4. Discussion

The present study described intakes of energy and nutrients in south-east Chinese people and presented the comparison of those between Chinese and Italian, Japanese and American diets in males and females for different age groups. There were marked differences in nutrients consumption between Chinese subjects and those from the other three countries. Firstly, the contribution of carbohydrate to energy in Chinese subjects was lower than that in Japanese and American subjects, but higher than that in Italian subjects; however, the energy contribution from fat in Chinese subjects was higher than that in Japanese and American subjects, and similar to that in Italian subjects. Secondly, the Chinese diet had lower daily intakes of fiber, calcium, phosphorus, potassium, selenium, vitamin A, vitamin B_1_, vitamin B_2_ and vitamin C, compared with the Japanese, American and Italian diets; nevertheless, intakes of sodium, iron, copper and vitamin E were higher among Chinese subjects relative to the people of other three countries.

### 4.1. Nutrients Intakes in Chinese People Changing Even Worse than Those in American People

Across countries, the predominant diets are clearly different and highly related with human health. Both the Italian and Japanese diets are known to be healthy, while the American diet is generally qualified as unhealthy [11]. The traditional Chinese diet includes rice, wheat and wheat products and vegetables with low animal-source foods [23]. It has been considered as extremely healthy diet, like the Italian and Japanese diets [14]. However, China seems to be rapidly relinquishing its traditional diet with its rapid economic development. China has experienced remarkable shifts in its disease patterns from the decreasing prevalence of malnutrition and nutrition deficiencies to a high prevalence of diet-related non-communicable diseases, such as obesity, diabetes, cardiovascular disease, and cancer [14]. In addition, China is also undergoing a remarkably fast, but undesirable shift towards a stage of nutrition transition dominated by a high intake of edible oils, processed foods and animal foods and a low intake of coarse grains, legumes and other healthy foods [2]. These shifts have been the basis for the change in nutrient intakes. The shifts of nutrient intakes accompanied by major cooking and eating behavior changes are leading to what might be characterized as an unhealthy Western diet. Therefore, we evaluated the dietary nutrients in south-east Chinese subjects and compared those to the healthy Italian, Japanese diets and unhealthy American diet. In the present study, 2659 participants aged 2.0–89.2 years were enrolled to evaluate the dietary nutrients in south-east Chinese subjects. We found that compared with the American subjects, Chinese subjects consumed lower intakes of fiber, carbohydrate and protein. Nevertheless, fat and cholesterol intakes in Chinese subjects were higher than those in American females, and similar to those in American males. Lower contribution of carbohydrate and protein and higher contribution of fat to energy in Chinese subjects than those in American subjects were also found. Therefore, macronutrients intakes of Chinese subjects had been close to those of American subjects, and many macronutrients were even worse than those of American subjects. Furthermore, the Chinese diet had lower daily intakes of calcium, phosphorus, potassium, selenium, vitamin A, vitamin B_1_, vitamin B_2_, vitamin C and niacin, and higher intakes of sodium, iron, copper and vitamin E, compared with the American diet. Therefore, these results demonstrated that nutrients intakes in Chinese people have been changing even worse than those in American people. The relative low prices of animal-source foods and oils partially explain these shifts and income increases are a second important cause [24,25]. In addition, another reason is that cooking and eating behaviors are changing rapidly, including the decrease in the percentage of food cooked in healthy ways (steamed, boiled, baked) and the increase in snacking, fried foods and away from-home food consumption [26].

### 4.2. Nutritional Transition of the Chinese Diet from Traditional to Non-Traditional Diet

Compared with the 2002 China National Nutrition and Health Survey data [27], intakes of total energy, carbohydrate, fiber, phosphorus, potassium, sodium, iron, copper, vitamin A, vitamin B_1_, vitamin B_2_, vitamin C and vitamin E decreased, while intakes of fat, calcium and selenium increased, with stable intake of protein. In addition, in the nine provinces in the China Health and Nutrition Survey, there were rapid changes in the percent energy intake from carbohydrate and fat between 1991 and 2011 [2]. The energy contributions from carbohydrate were 66.0%, 59.8% and 54.3% in 1991, 2000 and 2011, respectively; while the proportions of energy derived from fat were 21.8%, 27.8% and 32.0% in 1991, 2000 and 2011, respectively. Moreover, our study found that the percent energy from carbohydrate and fat in Chinese subjects ranged from 45.6% to 53.9% and from 33.2% to 41.0% in different age groups, respectively. Furthermore, Zhou *et al.* [28] once compared nutrient intakes of middle-aged men and women, aged 40–59 years, in Chinese, Japanese and American subjects in the late 1990s (1997–1999). This study showed higher fiber intake and percent energy from carbohydrate and lower cholesterol intake and percentage of energy from fat in Chinese subjects than those in Japanese and American subjects; these findings were consistent with another study carried out in women aged 40–70 years in Shanghai (China), Japan and America, from 1997 to 2000 [26]. Nevertheless, in our study we found lower fiber intake and percent energy from carbohydrate and higher percentage of energy from fat in Chinese subjects than those in Japanese and American subjects, which were opposite to the results in the late 1990s. In summary, these results demonstrated that fat intake has increased but the intake of complex carbohydrates and fiber has decreased in Chinese people in the past two decades. Therefore, the structure of the Chinese diet has been shifting away from the traditional diet toward high-fat, low-carbohydrate and low-fiber diets. However, it is noted that the older adults tended to retain the traditional diet (Supplementary Table S4).

### 4.3. Trends of Minerals and Vitamins

The truly healthy trends for Chinese diet were the reduction in intakes of sodium and increases in intakes of calcium. High sodium and low potassium intakes are the key risk factors for hypertension [29,30,31,32]. Although sodium intake decreased, it remained double those recommended by the Institute of Medicine and the WHO; and potassium intake was below the recommended amount [33]. Our findings were consistent with other studies [28,33]. However, it should be noted that dietary method is an inadequate assessment of sodium, and 24-h urine sodium is the only way to estimate sodium intake [34]. Nowadays, the major source of dietary sodium remains added salt, followed by soy sauce, processed foods, and monosodium glutamate [33]. Therefore, replacing sodium with potassium in salt is an option with the potential to control and prevent hypertension and improve the health of the Chinese population [33]. In addition, calcium intake increased in the past decade. Nevertheless, it remained far below the recommended amount [35]. Deficient intake of calcium from cereals, vegetables, legumes and dairy mainly accounted for the low intake of calcium [36]. Iron intake in males was higher than that recommended by the Chinese Nutrition Society [35], while it was comparable for females. However, the main source of iron intake was from plant-based foods among this study population, which has low bioavailability [37]. So iron intake might not meet the needs of the body, especially in females. Besides, vitamin B_2_ intake was inadequate. Its deficiency has been shown to negatively affect iron absorption and utilization, which is associated with increased risk of anemia [38]. Consequently, a higher intake of fruit, vegetables, cereals, legumes, dairy and certain animal-source foods should be recommended.

Zhou *et al.* [28] also compared intakes of minerals and vitamins in Chinese, Japanese and American people in the late 1990s. The compared results of calcium, sodium, phosphorus, potassium, selenium, magnesium, vitamin A and vitamin C intakes in this study were consistent with our results. However, this study showed similar intake of iron (between Chinese and American people) and vitamin E (between Chinese and Japanese, American people), while we found higher intakes of iron and vitamin E in Chinese people than those in Japanese and American people. Nevertheless, Chen *et al.* [26] reported higher intakes of iron and vitamin E in Chinese people than those in Japanese and American people in the late 1990s, which were consistent with our study. Together, these findings demonstrated that excessive, especially inadequate intakes of a range of minerals and vitamins have been persistent in the past decade.

### 4.4. Strengths and Limitations

To the best of our knowledge, this was the first study to compare of nutrient intakes between Chinese and Italian, Japanese and American diets in males and females for different age groups. However, our research has several limitations. First, as expected, in some age and sex groups, and in particular in children and teenagers, the sample size might be not sufficient. Second, energy and nutrient intakes in present study were estimated by three consecutive 24-h dietary recalls, which is frequently used in dietary assessment. We could not avoid the possibility of recall bias and other unknown confounding factors, which might influence the precise assessment of nutrient intakes. However, the average intake over three days can offer a relatively valid estimate of usual nutrient intakes [39,40]. In addition, the diet data collected between August and October of each year may not reflect seasonal differences in nutrients consumption. Third, it is noted that our present results were without adjustment for sampling weight, which might limit the ability to generalize our findings to the general population. Finally, because of possible differences in sampling frame, dietary assessment methods (24-h dietary recall or dietary record) and data-processing methods, a detailed cross-country comparison of nutritional data would introduce bias. However, Kroes *et al.* [41] found a similar effect on estimating dietary intake between 24-h dietary recall and dietary record. Moreover, it was clear that the nutrient intakes of the Chinese people in this study were quite different from those found in the people of other three countries, regardless of nutrients adjusted for energy.

## 5. Conclusions

The present study demonstrated that the structure of the Chinese diet has been shifting away from the traditional diet toward high-fat, low-carbohydrate and low-fiber diets, and nutrients intakes in Chinese people have been changing even worse than those in American people. From a public health perspective, the present findings imply that a healthy diet should be advocated in the Chinese people.

## Authors Contributions

Yunxian Yu developed the initial idea and designed the study. Zhaopin Wang, Biao Zhou, Lijuan Wang, Lichun Huang, Shuying Jiang, Zeyu Liu and Jingxin Jiang were responsible for the data and sample collection. Ronghua Zhang and Biao Zhou was responsible for the quality control. Ying Fei and Shuangshuang Zheng conducted the statistical analysis. Ronghua Zhang and Zhaopin Wang wrote the manuscript. All authors contributed to the interpretation of the results and were involved in preparing the final manuscript.

## Supplementary Files

Supplementary File 1
