# Supplementary material for: The Difference in Nutrient Intakes between Chinese and Mediterranean, Japanese and American Diets
Source: Nutrients. 2015 Jun 9;7(6):4661–88. doi: 10.3390/nu7064661 (PMC4488807; doi:10.3390/nu7064661)
Supplement: Supplementary File 1 [file nutrients-07-04661-s001.docx]

**Supplementary Information**

**Table S1.** The distributions of energy and nutrient intakes of study subjects by sex in Chinese.

| **Energy and Nutrients** | **Males  (*N* = 1274, Mean ± SD)** | **Females  (*N* = 1385, Mean ± SD)** | ***p* value** |
| --- | --- | --- | --- |
| **Total energy, kcal** | 2194.78 ± 856.59 | 1766.14 ± 716.33 | < 0.0001 |
| **Fat, g** | 89.06 ± 45.12 | 72.73 ± 37.10 | < 0.0001 |
| **Fat, %** | 36.46 ± 11.15 | 37.03 ± 11.39 | 0.1892 |
| **Protein, g** | 73.01 ± 30.73 | 61.89 ± 28.24 | < 0.0001 |
| **Protein, %** | 13.62 ± 3.51 | 14.27 ± 3.88 | < 0.0001 |
| **Carbohydrate, g** | 261.47 ± 119.60 | 219.11 ± 112.56 | < 0.0001 |
| **Carbohydrate, %** | 48.06 ± 12.34 | 49.44 ± 12.41 | 0.0041 |
| **Cholesterol, mg** | 323.10 ± 235.38 | 289.91 ± 224.34 | 0.0002 |
| **Fiber, g** | 10.67 ± 7.63 | 10.21 ± 7.86 | 0.1238 |
| **Calcium, mg** | 448.08 ± 240.14 | 405.27 ± 234.89 | < 0.0001 |
| **Phosphorus, mg** | 1001.43 ± 405.63 | 853.91 ± 372.75 | < 0.0001 |
| **Potassium, mg** | 1739.00 ± 819.69 | 1561.75 ± 776.17 | < 0.0001 |
| **Sodium, mg** | 6060.84 ± 4838.47 | 5292.83 ± 4968.56 | < 0.0001 |
| **Iron, mg** | 21.97 ± 10.78 | 19.23 ± 14.19 | < 0.0001 |
| **Zinc, mg** | 11.48 ± 4.63 | 9.57 ± 4.35 | < 0.0001 |
| **Copper, mg** | 2.11 ± 1.17 | 1.86 ± 1.21 | < 0.0001 |
| **Selenium, ug** | 48.79 ± 30.33 | 42.30 ± 29.89 | < 0.0001 |
| **Manganese, mg** | 5.95 ± 3.49 | 5.03 ± 2.68 | < 0.0001 |
| **Vitamin A, μg REs ^&^** | 475.84 ± 682.87 | 420.42 ± 451.78 | 0.0145 |
| **Vitamin B_1_, mg** | 0.98 ± 0.55 | 0.75 ± 0.37 | < 0.0001 |
| **Vitamin B_2_, mg** | 0.85 ± 0.43 | 0.72 ± 0.39 | < 0.0001 |
| **Vitamin C, mg** | 63.92 ± 58.84 | 62.85 ± 47.52 | 0.6034 |
| **Vitamin E, mg** | 30.89 ± 20.01 | 26.58 ± 15.68 | < 0.0001 |

^&^ REs: Retinol equivalents.

**Table S2.** Comparison of daily intake of nutrients ^#^ adjusted for energy by age in children^ and males between Chinese and Italian.

| **Energy and Nutrients** | **Children (3–9 Years)** | | **Males (10–17 Years)** | | **Males (18–64 Years)** | | **Males (65 Years and above)** | |
| --- | --- | --- | --- | --- | --- | --- | --- | --- |
|  | **Chinese  (*N* = 112)** | **Italian  (*N* = 193)** | **Chinese  (*N* = 53)** | **Italian  (*N* = 108)** | **Chinese  (*N* = 896)** | **Italian  (*N* = 1068)** | **Chinese  (*N* = 263)** | **Italian  (*N* = 202)** |
| **Total energy, kcal** | 1322 ± 561 | 1914 ± 488 * | 1865 ± 700 | 2576 ± 744 * | 2330 ± 865 | 2390 ± 650 * | 1981 ± 740 | 2296 ± 556 * |
| **Total energy, MJ** | 5.5 ± 2.3 | 8.0 ± 2.0 * | 7.8 ± 2.9 | 10.8 ± 3.1 * | 9.7 ± 3.6 | 10.0 ± 2.7 * | 8.3 ± 3.1 | 9.6 ± 2.3 * |
| **Cholesterol, mg/MJ** | 53.5 ± 39.9 | 36.0 ± 13.0 * | 37.4 ± 22.9 | 32.6 ± 9.0 | 36.7 ± 26.4 | 33.0 ± 11.9 * | 36.0 ± 26.2 | 31.3 ± 11.8 * |
| **Fiber, g/MJ** | 1.0 ± 0.6 | 1.8 ± 0.5 * | 1.1 ± 0.6 | 1.7 ± 0.5 * | 1.2 ± 0.7 | 2.0 ± 0.6 * | 1.4 ± 0.9 | 2.3 ± 0.8 * |
| ***Minerals*** |  |  |  |  |  |  |  |  |
| **Potassium, mg/MJ** | 194 ± 59 | 310 ± 62 * | 196 ± 83 | 296 ± 61 * | 191 ± 62 | 329 ± 75 * | 212 ± 75 | 348 ± 71 * |
| **Phosphorus, mg/MJ** | 114 ± 24 | 150 ± 26 * | 112 ± 24 | 139 ± 21 * | 111 ± 27 | 140 ± 22 * | 118 ± 31 | 140 ± 21 * |
| **Calcium, mg/MJ** | 55 ± 22 | 96 ± 30 * | 50 ± 30 | 83 ± 25 * | 50 ± 24 | 80 ± 27 * | 58 ± 27 | 86 ± 28 * |
| **Magnesium, mg/MJ** | 30.1 ± 7.6 | 29.3 ± 7.2 | 29.4 ± 7.6 | 27.2 ± 5.8 * | 31.6 ± 8.4 | 31.1 ± 7.5 * | 35.0 ± 10.1 | 31.3 ± 7.4 * |
| **Iron, mg/MJ** | 2.4 ± 1.0 | 1.2 ± 0.3 * | 2.4 ± 0.9 | 1.2 ± 0.3 * | 2.5 ± 1.0 | 1.3 ± 0.3 * | 2.6 ± 1.1 | 1.4 ± 0.3 * |
| **Zinc, mg/MJ** | 1.2 ± 0.3 | 1.3 ± 0.3 * | 1.3 ± 0.3 | 1.2 ± 0.2 | 1.3 ± 0.3 | 1.3 ± 0.3 | 1.3 ± 0.3 | 1.3 ± 0.2 * |
| ***Vitamins*** |  |  |  |  |  |  |  |  |
| **Vitamin A, REs ^&^ μg/MJ** | 58 ± 76 | 93 ± 111 * | 46 ± 44 | 76 ± 82 * | 54 ± 83 | 92 ± 116 * | 54 ± 51 | 95 ± 94 * |
| **Vitamin B_1_, mg/MJ** | 0.10 ± 0.02 | 0.12 ± 0.03 * | 0.10 ± 0.03 | 0.12 ± 0.03 * | 0.11 ± 0.04 | 0.11 ± 0.03 * | 0.10 ± 0.04 | 0.11 ± 0.03 * |
| **Vitamin B_2_, mg/MJ** | 0.10 ± 0.04 | 0.18 ± 0.05 * | 0.10 ± 0.04 | 0.16 ± 0.04 * | 0.09 ± 0.03 | 0.16 ± 0.04 * | 0.10 ± 0.04 | 0.16 ± 0.04 * |
| **Vitamin C, mg/MJ** | 5.9 ± 4.5 | 13.5 ± 7.6 * | 9.2 ± 12.2 | 13.0 ± 8.6 * | 7.0 ± 5.4 | 13.0 ± 8.3 * | 9.2 ± 6.3 | 13.7 ± 8.7 * |
| **Vitamin E, mg/MJ** | 3.5 ± 1.7 | 1.3 ± 0.3 * | 3.0 ± 1.5 | 1.3 ± 0.4 * | 3.4 ± 1.7 | 1.4 ± 0.4 * | 3.3 ± 1.6 | 1.4 ± 0.4 * |

^#^ Mean ± SD; ^&^ REs: Retinol equivalents; ^ Two infants (< 3 years) were not included, with males and females grouped in the case of children; * *p* < 0.04 after FDR adjustment, *versus* Chinese.

**Table S3.** Comparison of daily intake of nutrients^#^ adjusted for energy by age in females between Chinese and Italian.

| **Energy and Nutrients** | **Females (10–17 Years)** | | **Females (18–64 Years)** | | **Females (65 Years and above)** | |
| --- | --- | --- | --- | --- | --- | --- |
|  | **Chinese (*N* = 75)** | **Italian (*N* = 139)** | **Chinese (*N* = 1027)** | **Italian (*N* = 1245)** | **Chinese (*N* = 231)** | **Italian (*N* = 316)** |
| **Total energy, kcal** | 1535 ± 527 | 2091 ± 532 * | 1845 ± 750 | 1939 ± 526 * | 1613 ± 558 | 1834 ± 486 * |
| **Total energy, MJ** | 6.4 ± 2.2 | 8.7 ± 2.2 * | 7.7 ± 3.1 | 8.1 ± 2.2 * | 6.7 ± 2.3 | 7.7 ± 2.0 * |
| **Cholesterol, mg/MJ** | 46.3 ± 39.9 | 35.2 ± 11.4 * | 41.1 ± 30.1 | 32.6 ± 12.8 * | 35.8 ± 29.3 | 31.7 ± 11.8 * |
| **Fiber, g/MJ** | 1.1 ± 0.7 | 1.9 ± 0.5 * | 1.4 ± 0.8 | 2.2 ± 0.7 * | 1.5 ± 0.8 | 2.5 ± 0.7 * |
| ***Minerals*** |  |  |  |  |  |  |
| **Potassium, mg/MJ** | 197 ± 69 | 319 ± 79 * | 214 ± 71 | 362 ± 90 * | 226 ± 78 | 376 ± 95 * |
| **Phosphorus, mg/MJ** | 114 ± 29 | 145 ± 24 * | 117 ± 29 | 146 ± 24 * | 123 ± 32 | 148 ± 28 * |
| **Calcium, mg/MJ** | 55 ± 28 | 89 ± 27 * | 56 ± 27 | 91 ± 30 * | 62 ± 31 | 100 ± 36 * |
| **Magnesium, mg/MJ** | 31.7 ± 8.8 | 29.1 ± 9.1 | 34.2 ± 9.6 | 32.5 ± 8.5 * | 36.9 ± 10.9 | 32.6 ± 9.0 * |
| **Iron, mg/MJ** | 2.3 ± 0.8 | 1.2 ± 0.3 * | 2.6 ± 1.3 | 1.3 ± 0.3 * | 2.8 ± 1.4 | 1.3 ± 0.3 * |
| **Zinc, mg/MJ** | 1.3 ± 0.3 | 1.3 ± 0.3 * | 1.3 ± 0.3 | 1.3 ± 0.3 | 1.3 ± 0.3 | 1.3 ± 0.3 |
| ***Vitamins*** |  |  |  |  |  |  |
| **Vitamin A, REs ^&^ μg/MJ** | 51 ± 38 | 88 ± 110 * | 58 ± 55 | 104 ± 121 * | 63 ± 66 | 106 ± 90 * |
| **Vitamin B_1_, mg/MJ** | 0.10 ± 0.03 | 0.12 ± 0.03 | 0.10 ± 0.03 | 0.12 ± 0.03 * | 0.10 ± 0.03 | 0.11 ± 0.03 * |
| **Vitamin B_2_, mg/MJ** | 0.10 ± 0.04 | 0.17 ± 0.04 * | 0.10 ± 0.04 | 0.17 ± 0.05 * | 0.10 ± 0.04 | 0.18 ± 0.05 * |
| **Vitamin C, mg/MJ** | 7.6 ± 5.7 | 14.9 ± 11.1 * | 8.8 ± 5.9 | 15.6 ± 9.5 * | 10.1 ± 7.0 | 17.0 ± 11.9 * |
| **Vitamin E, mg/MJ** | 3.9 ± 1.9 | 1.4 ± 0.4 * | 3.6 ± 1.7 | 1.5 ± 0.4 * | 3.5 ± 1.6 | 1.4 ± 0.4 * |

^#^ Mean ± SD; ^&^ REs: Retinol equivalents; **p* < 0.04 after FDR adjustment, *versus* Chinese.

**Table S4.** Comparison of daily intake of nutrients adjusted for energy by age in Chinese.

| **Energy and Nutrients** | **Children (1–19 Years)  (*N* = 260, Mean ± SD)** | **Adults (20–59 Years)  (*N* = 1596, Mean ± SD)** | **Older Adults (60 Years and above)  (*N* = 803, Mean ± SD)** | ***p* value** |
| --- | --- | --- | --- | --- |
| **Total energy, kcal** | 1532 ± 635 | 2089 ± 819 | 1880 ± 800 | < 0.0001 |
| **Total energy, MJ** | 6.4 ± 2.7 | 8.7 ± 3.4 | 7.9 ± 3.4 | < 0.0001 |
| **Fat, g/MJ** | 10.4 ± 3.1 | 10.0 ± 3.0 | 9.2 ± 3.0 | < 0.0001 |
| **Protein, g/MJ** | 8.3 ± 2.1 | 8.3 ± 2.2 | 8.5 ± 2.2 | 0.0576 |
| **Carbohydrate, g/MJ** | 28.6 ± 7.5 | 28.7 ± 7.3 | 30.2 ± 7.5 | < 0.0001 |
| **Cholesterol, mg/MJ** | 48.1 ± 36.7 | 39.2 ± 28.8 | 36.5 ± 27.4 | < 0.0001 |
| **Fiber, g/MJ** | 1.1 ± 0.7 | 1.3 ± 0.8 | 1.5 ± 0.9 | < 0.0001 |
| ***Minerals*** |  |  |  |  |
| **Calcium, mg/MJ** | 54.0 ± 25.2 | 52.2 ± 25.3 | 58.7 ± 28.1 | < 0.0001 |
| **Phosphorus, mg/MJ** | 113.8 ± 25.3 | 113.2 ± 28.4 | 120.1 ± 29.8 | < 0.0001 |
| **Potassium, mg/MJ** | 196.9 ± 67.0 | 200.9 ± 67.7 | 217.6 ± 73.9 | < 0.0001 |
| **Sodium, mg/MJ** | 637.5 ± 356.0 | 699.8 ± 485.3 | 726.1 ± 471.4 | 0.0299 |
| **Manganese, mg/MJ** | 30.55 ± 7.85 | 32.51 ± 8.93 | 35.74 ± 10.24 | < 0.0001 |
| **Iron, mg/MJ** | 2.36 ± 0.88 | 2.53 ± 1.14 | 2.70 ± 1.21 | < 0.0001 |
| **Zinc, mg/MJ** | 1.25 ± 0.28 | 1.29 ± 0.32 | 1.33 ± 0.29 | 0.0012 |
| **Copper, mg/MJ** | 0.24 ± 0.14 | 0.24 ± 0.15 | 0.25 ± 0.14 | 0.2367 |
| **Selenium, ug/MJ** | 5.60 ± 3.22 | 5.65 ± 3.33 | 5.89 ± 3.41 | 0.2157 |
| ***Vitamins*** |  |  |  |  |
| **Vitamin A, REs ^&^ μg/MJ** | 53.13 ± 57.78 | 56.15 ± 72.60 | 57.92 ± 55.67 | 0.5865 |
| **Vitamin B_1_, mg/MJ** | 0.10 ± 0.03 | 0.11 ± 0.04 | 0.10 ± 0.03 | 0.0009 |
| **Vitamin B_2_, mg/MJ** | 0.10 ± 0.04 | 0.10 ± 0.04 | 0.10 ± 0.04 | 0.0589 |
| **Niacin, mg/MJ** | 1.74 ± 0.62 | 1.88 ± 0.64 | 1.91 ± 0.64 | 0.0009 |
| **Vitamin C, mg/MJ** | 7.25 ± 7.28 | 7.72 ± 5.53 | 9.45 ± 6.63 | < 0.0001 |
| **Vitamin E, mg/MJ** | 3.46 ± 1.73 | 3.53 ± 1.66 | 3.46 ± 1.65 | 0.6100 |

^&^ REs: Retinol equivalents.

© 2015 by the authors; licensee MDPI, Basel, Switzerland. This article is an open access article distributed under the terms and conditions of the Creative Commons Attribution license (http://creativecommons.org/licenses/by/4.0/).
